# Treatment efficacy in a metastatic small intestinal neuroendocrine tumour grade 2 cohort

**DOI:** 10.1530/ERC-22-0316

**Published:** 2023-02-14

**Authors:** Dimitrios Papantoniou, Malin Grönberg, Espen Thiis-Evensen, Halfdan Sorbye, Kalle Landerholm, Staffan Welin, Eva Tiensuu Janson

**Affiliations:** 1 Department of Medical Sciences, Endocrine Oncology, Uppsala University, Uppsala, Sweden; 2 Department of Oncology, Ryhov County Hospital, Jönköping, Sweden; 3 Oslo University Hospital, Rikshospitalet, Deptartment of Organ Transplant, Oslo, Norway; 4 Haukeland Hospital, Deptartment of Oncology, Bergen, Norway; 5 University of Bergen, Deptartment of Clinical Medicine, Bergen, Norway; 6 Department of Biomedical and Clinical Sciences, Linköping University, Linköping, Sweden; 7 Department of Surgery, Ryhov County Hospital, Jönköping, Sweden

**Keywords:** small intestinal neuroendocrine tumours, Si-NET, grade 2, somatostatin analogues, interferon, PRRT, peptide receptor radionuclide treatment, Ki-67, somatostatin receptor negative

## Abstract

Small intestinal neuroendocrine tumours (Si-NET) are often studied as a uniform group. Proliferation index Ki-67 influences prognosis and determines tumour grade. We hypothesized that Si-NET grade 2 (G2) tumours, which have a higher Ki-67 than G1 tumours, might benefit less from established treatments for metastatic disease. We conducted a retrospective cohort study of 212 patients with metastatic Si-NET G2 treated in two Swedish hospitals during 20 years (2000–2019). Median cancer-specific survival on first-line somatostatin analogues (SSA) was 77 months. Median progression-free survival (PFS) was 12.4 months when SSA was given as monotherapy and 19 months for all patients receiving first-line SSA. PFS after SSA dose escalation was 6 months in patients with radiological progression. Treatment efficacies of SSA and peptide receptor radionuclide treatment (PRRT) were studied separately in patients with Ki-67 of 3–5%, 5–10% and 10–20%. For SSA, PFS was significantly shorter at higher Ki-67 levels (31, 18 and 10 months, respectively), while there was only a minor difference in PFS for PRRT (29, 25 and 25 months). Median PFS for sequential treatment with interferon-alpha (IFNα), everolimus and chemotherapy was 6, 5 and 9 months. IFNα seemed to be effective in tumours with low somatostatin–receptor expression. In conclusion, established treatments appeared effective in Si-NET G2, despite their higher proliferation index compared to G1 tumours. However, efficacy of SSA but not PRRT was reduced at higher Ki-67 levels. SSA dose escalation provided limited disease stabilization.

## Introduction

Small intestinal neuroendocrine tumours (Si-NET) are grouped according to their proliferation index (Ki-67) into grade 1 (G1, Ki-67 <3%), grade 2 (G2, Ki-67 3–20%) and grade 3 (G3, Ki-67 >20%) (Klimstra *et al.* 2019). Ki-67 has been reported to correlate with prognosis as a continuous variable (Panzuto *et al.* 2014*a*, Bertani *et al.* 2015, Lamarca *et al.* 2019) and at various standard (Panzuto *et al.* 2012, Araujo *et al.* 2013, Panzuto *et al.* 2014*a*, Landerholm & Falkmer 2015, Faggiano *et al.* 2016, Özaslan *et al.* 2016, Lamarca *et al.* 2019) and alternative (Palazzo *et al.* 2013, Ezziddin *et al.* 2014, Panzuto *et al.* 2014*a*, Faggiano *et al.* 2016, Sun *et al.* 2018, Aalbersberg *et al.* 2019) cut-offs in mostly mixed NET cohorts. Four studies, of which only one included predominantly Si-NET patients, have examined the effect of G2 Ki-67 levels on somatostatin analogue (SSA) or peptide radionuclide receptor therapy (PRRT) treatment efficacy. Median progression-free survival (PFS) was shorter for SSA at Ki-67 >5%, while differences were less prominent for PRRT, with the larger study detecting shorter median overall survival (OS) only in patients with Ki-67 >10% (Palazzo *et al.* 2013, Ezziddin *et al.* 2014, Faggiano *et al.* 2016, Aalbersberg *et al.* 2019).

SSA, everolimus and PRRT are registered for treatment of metastatic Si-NET (Janson *et al.* 2021), based on four prospective trials: PROMID and CLARINET evaluated long-acting SSA compared to placebo. PROMID included treatment-naïve G1 tumours; median time to tumour progression (TTP) favoured the SSA group at 14 vs 6 months (Rinke *et al.* 2009). CLARINET, which included non-functioning GEP-NEN with Ki-67 <10%, showed a 2-year PFS of 65% vs 33% in favour of SSA (Caplin *et al.* 2014). RADIANT-4 showed longer median PFS for everolimus compared to placebo (11 vs 4 months) in non-functioning NET of mixed origin including one-third G2 tumours (Yao *et al.* 2016). NETTER-1 showed higher 20-month PFS rate (65% vs 11%) for PRRT compared with an above-label dose of SSA in progressive somatostatin receptor (SSTR)-positive Si-NET (30% G2 tumours) (Strosberg *et al.* 2017). Median OS was 48 and 36 months, respectively (Strosberg *et al.* 2021).

Three small randomized trials comparing SSA vs SSA plus interferon-alpha (IFNα) showed some advantage for the combination but could not detect a statistically significant OS benefit (Fazio *et al.* 2007), while two recent network meta-analyses confirmed the efficacy of SSA plus IFNα in non-pancreatic NET (Kaderli *et al.* 2019, Walter *et al.* 2021).

Tumours with low SSTR expression remain a therapeutic challenge, as PRRT is not efficient in this population, everolimus is only approved for non-functional Si-NET and SSA is less documented. A propensity score-matched analysis of SSTR-negative and SSTR-positive patients showed that SSTR-negative patients had shorter median OS, even after correcting for grade and that treatment with SSA did not improve prognosis (Refardt *et al.* 2020).

Increase of SSA dose is often used as a first step after progression of Si-NET on first-line SSA, based on retrospective publications (Ferolla *et al.* 2012, Strosberg 2014, Lamberti *et al.* 2020, Diamantopoulos *et al.* 2021). A prospective phase 2 trial (CLARINET FORTE) with above-label dose of lanreotide autogel recently reported moderate efficacy, with a median PFS of 8.3 months in the Si-NET subgroup. Only 22 of 51 patients had Ki-67 >2% and four patients had Ki-67 >10%; the latter had a median PFS of 5.5 months (Pavel *et al.* 2021).

Pivotal studies have either excluded Si-NET G2 or grouped them together with the much more frequent G1 tumours, often with NET of other origin. Within the Si-NET group, G1 tumours are three times as frequent as G2 tumours (Snorradottir *et al.* 2022). Few retrospective series focus on Si-NET, and only one presents solely G2 tumours (Papantoniou *et al.* 2021). As Ki-67 possibly impacts treatment outcome (Arnold *et al.* 2005, Palazzo *et al.* 2013, Ezziddin *et al.* 2014, Panzuto *et al.* 2014*b*, Albertelli *et al.* 2021), we hypothesized a lower efficacy in G2 tumours. We hereby evaluated the effect of medical treatments in a large cohort of exclusively Si-NET G2.

## Materials and methods

In this retrospective cohort study, all 212 patients with metastatic Si-NET G2 diagnosed between 2000 and 2019 and receiving any treatment at the Department of Endocrine Oncology, Uppsala University Hospital, a tertiary referral centre, and at the Department of Oncology, Ryhov County Hospital, a regional hospital, were eligible for inclusion. One-third of the patients were referred from other hospitals in Sweden and Norway. Patients with radical surgical resection not relapsing during the study period were not included. Following approval from the Uppsala ethical review board, data on patients’ clinical status including Eastern Cooperative Oncology Group performance status (ECOG PS), treatments given, Ki-67, laboratory tests, radiology and cause of death were extracted from the hospitals’ medical records. Chromogranin A (CgA) and 5-hydroxyindoleacetic acid (5-HIAA) were reported as times the upper limit of normal. In case of multiple biopsies, the highest Ki-67 value before or within 6 months of the start of a new line of treatment was reported. SSTR status was evaluated on Octreoscan in the majority of patients; during the final years of the study, ^68^Gallium DOTATATE positron emission tomography could alternatively be used. An uptake below or equal to liver uptake was considered negative/low. Cases with small tumours not visible on the initial Octreoscan, which upon progression showed clear uptake in subsequent imaging, were considered positive. Survival status was censored on October 31, 2021, or at last known contact. Causes of death due to tumour progression, adverse events, surgical morbidity and cases where cause of death was indeterminate but cancer-related death likely were classified as cancer-specific mortality. Patients dying from causes unrelated to their NET tumour were censored at the time of death.

Treatments studied included SSA, PRRT, everolimus, IFNα and chemotherapy. Patients were treated with various doses of SSA. In the first study years, treatment was often initiated at lower (hereby referred to as below-label) doses, mostly 20 mg of octreotide long-acting release (LAR) every 4 weeks. After the publication of PROMID and CLARINET trials, a standard (label) dose of 30 mg octreotide LAR/120 mg lanreotide autogel every 4 weeks was used. Dose could be escalated to above-label doses, often in consecutive steps, for progression or symptom control.

Cancer-specific survival (CSS) and OS for first-line treatment were calculated from start of treatment for metastatic disease to cancer-related death or death from any cause, respectively. PFS was calculated from start of each treatment to radiological progression, unequivocal clinical progression or death. Radiological progression was based on conventional multidisciplinary team assessment at 3- to 6-month intervals and defined as any unequivocal increase in the size of known tumours or detection of new lesions. Biochemical partial response was defined as a reduction of baseline CgA or 5-HIAA by at least 50% and biochemically progressive disease (PD) as an increase by at least 25%, whereas values in between were deemed as biochemically stable disease (SD).

## Statistical methods

Statistical analysis was performed with R version 4.1.2 (R Foundation for Statistical Computing, Vienna, Austria) and the compareGroups package 4.0.0, using standard methodology (chi-square test for dichotomous variables, *t*-test or Kruskal–Wallis test for continuous variables and semi-parametric cox models for censored variables). Ki-67 was analysed both as a continuous non-linear variable, using restricted cubic splines (transformations of a variable which allow for summarizing relationships expected to be non-linear) (Greenland 1995) with three degrees of freedom in Cox models, and as a categorical variable in ≤5%, >5–10% and >10% groups. PFS and CSS were analysed using the Kaplan–Meier method, and between‐group differences were evaluated using a log‐rank test. Hazard ratios (HRs) and confidence intervals (CIs) were estimated from the Cox proportional hazards model. Adjusted survival curves, which represent expected survival curves corrected for covariates on the basis of a Cox model, were created with the survminer package 0.4.9 (Therneau *et al.* 2015, Biecek *et al.* 2023).

All tests were two-sided. *P* values <0.05 were considered statistically significant.

## Results

### Patients and treatments

**Figure 1:**
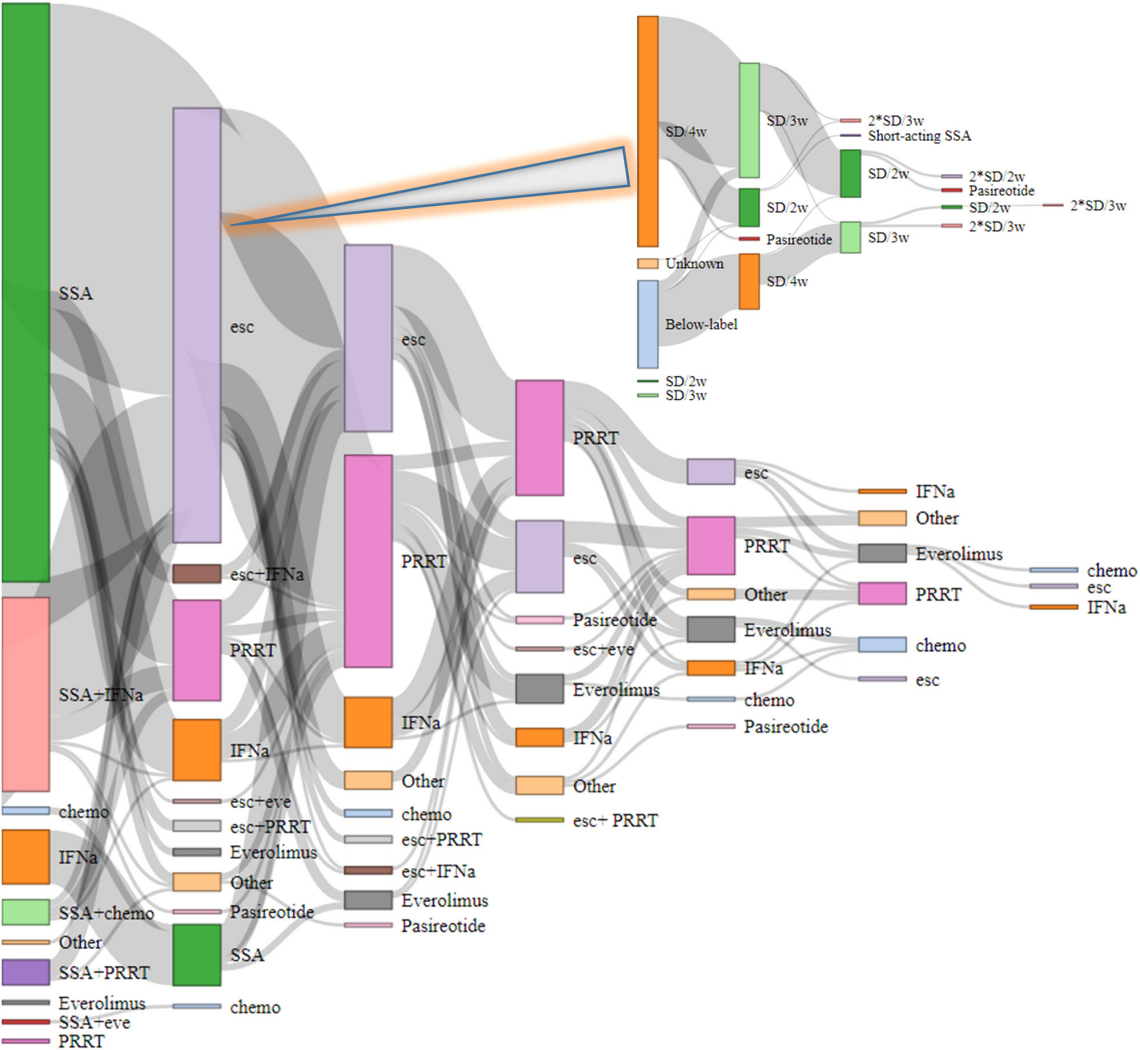
Treatment sequencing for all medical treatments and SSA dose escalations. Each vertical column represents a line of treatment; the height of each node is proportional to the number of patients treated, and the width of links between nodes to the number of patients transitioning between consecutive lines of treatment. SSA, somatostatin analogues; esc, escalation of SSA; IFNα, interferon-alpha; PRRT, peptide receptor radionuclide therapy; eve, everolimus; chemo, chemotherapy; SD, standard dose of SSA (30 mg octreotide LAR/120 mg lanreotide autogel); w, weeks.

Among 212 patients with Si-NET G2, 85 (40%) were female. The median age at treatment start was 65 (IQR 58–72) years. Surgical resection of the primary tumour, either with curative intention or for local symptom control, as per local standards at the time of the study, was performed in 151 cases (71%). Ki-67 was 3–5% in 72 (35%), 5–10% in 88 (42%) and 10–20% in 48 cases (23%) (Table 1). Two hundred and ten patients (99%) were treated with SSA, 95 (45%) with IFNα (with additionally nine cases of retreatment), 29 (14%) with everolimus, 17 (8%) with chemotherapy and 116 (55%) with PRRT (with additionally 25 cases of retreatment). Treatment sequencing is shown in Fig. 1.

**Table 1:** Baseline treatment characteristics.

	All	SSA	PRRT	IFNα	Everolimus	Chemotherapy
	*n* = 212	*n* = 210	*n* = 141^a^	*n*= 104^b^	*n* = 29	*n* = 17
Sex, *n* (%): Female	85 (40%)	84 (40%)	52 (37%)	42 (40%)	13 (45%)	6 (35%)
Age, median (IQR)	65 (58–72)	65 (58–72)	67 (60–73)	61 (54–69)	70 (63–72)	67 (54–72)
Performance status, *n* (%)						
0	82 (58%)	79 (56%)	68 (50%)	36 (54%)	4 (31%)	2 (29%)
1	40 (28%)	43 (30%)	48 (36%)	22 (33%)	5 (38%)	2 (29%)
≥2	20 (14%)	20 (14%)	19 (14%)	9 (13%)	4 (31%)	3 (43%)
Ki-67 (%), median (IQR)	7 (4–10)	7 (4–10)	8 (5–12)	6 (4–9)	8 (6–11)	9 (8–15)
Ki-67 (%), *n* (%)						
3–5	72 (35%)	71 (34%)	38 (27%)	39 (39%)	6 (24%)	2 (12%)
5–10	88 (42%)	87 (42%)	61 (43%)	46 (46%)	12 (48%)	7 (44%)
10–20	48 (23%)	49 (24%)	42 (30%)	14 (14%)	7 (28%)	7 (44%)
Liver metastases, *n* (%)	162 (76%)	163 (78%)	135 (96%)	80 (77%)	16 (55%)	13 (76%)
Line of treatment, *n* (%)						
1		196 (93%)	10 (7%)	60 (58%)	3 (10%)	9 (53%)
2		14 (7%)	76 (54%)	34 (33%)	4 (14%)	2 (12%)
≥3		0 (0%)	55 (39%)	9 (9%)	22 (76%)	6 (35%)
Start in combination, *n* (%)		68 (33%)	11 (9%)	50 (51%)	1 (6%)	7 (50%)

Baseline characteristics for all patients at first-line treatment start and per treatment given, irrespective of line. Percentages reported on patients with available data.

^a^One hundred sixteen primary treatments, 25 retreatments; ^b^95 cases of primary treatment, 9 retreatments.

IFNα, interferon-alpha; IQR, interquartile range; PRRT, peptide receptor radionuclide treatment; SSA, somatostatin analogues.

### SSA

SSA was the first-line treatment in 196 SSA-treated patients (93%). SSA was administered as monotherapy (*n* = 140, of which 126 at first line) or concomitantly with another drug, most often IFNα (*n* = 68). In two cases, sequencing was unknown. Median CSS and OS from start of first-line SSA was 77 and 70 months. Median PFS was 12.4 months for treatment with SSA monotherapy and 19 months for all patients treated with SSA at first line. Four patients (2%) discontinued SSA for gastrointestinal and liver toxicity. Patients with ECOG PS 0, 1 and ≥2 had a median CSS of 92, 91 and 24 months and a median PFS of 28, 25 and 6 months, respectively.

### Starting dose of SSA

**Figure 2:**
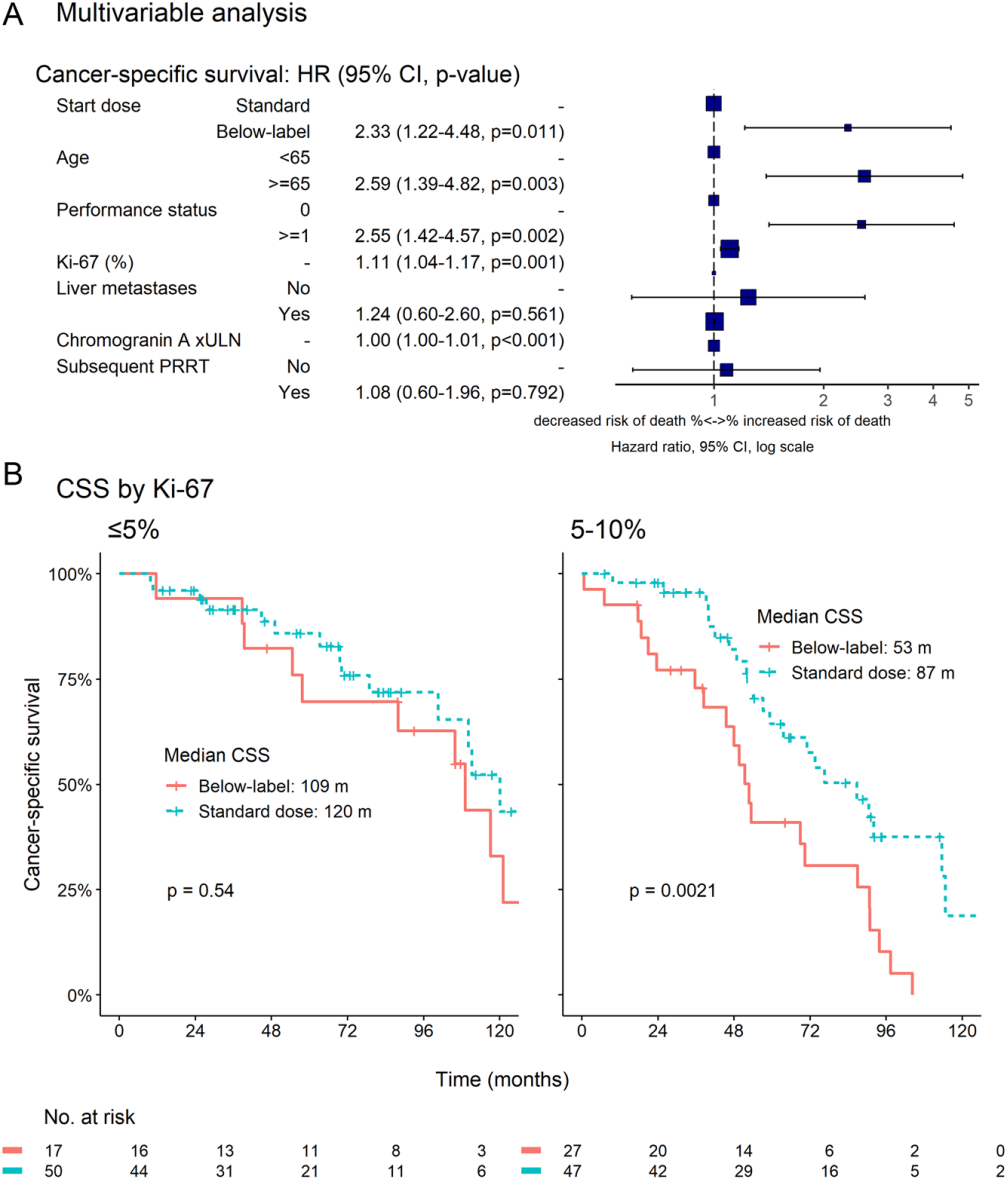
(A) Multivariable analysis of prognostic factors for cancer-specific survival (CSS) after initiation of treatment with first-line somatostatin analogues at below-label or standard doses. Chromogranin A is expressed as times the upper limit of normal (×ULN). A lower starting dose, increasing Ki-67 and chromogranin A, age ≥65 years and a performance status ≥1 are associated with higher risk of cancer-specific death. (B) CSS for patients with Ki-67 ≤5% and 5–10%. Only the 5–10% subgroup seems to benefit from the higher starting dose. Patients with Ki-67 >10% were not formally analysed, as only four patients were treated with a below-label dose in this subgroup. HR, hazard ratio; CI, confidence interval; PRRT, peptide receptor radionuclide therapy. A full colour version of this figure is available at https://doi.org/10.1530/ERC-22-0316.

Starting SSA dose was increased from below-label dose (often 20 mg octreotide LAR every 4 weeks) to standard dose (30 mg octreotide LAR/120 mg lanreotide autogel every 4 weeks) midway through the study period. We hypothesized that starting at a below-label dose and escalating at a later time point might have a negative impact on survival. The two groups had comparable baseline characteristics. In an unadjusted analysis, there was no significant CSS difference between patients starting at below-label dose (*n* = 48) and standard dose (*n* = 141) (69 vs 81 months, HR = 1.28, 95% CI 0.86–1.93, *P* = 0.23). After adjusting, though, for age, Ki-67, liver metastases, CgA, PS and subsequent PRRT use, CSS was shorter in the below-label dose group (HR = 2.33, 95% CI 1.22–4.48, *P* = 0.01, Fig. 2A).

In the subgroup of patients with Ki-67 5–10% (*n* = 74), median CSS was significantly shorter for patients treated with below-label doses of SSA (53 vs 87 months, *P* = 0.002). No difference was observed in the Ki-67 ≤5% group (*n* = 67, 109 vs 120 months, *P* = 0.54). Median CSS did not seem to differ for patients with Ki-67 >10% (62 vs 49 months), but this subgroup was not formally analysed, as only four patients were treated with a below-label dose (Fig. 2B).

PFS on initial treatment dose with SSA monotherapy did not differ significantly between the two groups (7 vs 14 months, HR = 1.30, 95% CI 0.76–2.22, *P* = 0.34). A trend to shorter PFS was seen for patients with Ki-67 5–10% treated with below-label doses (6 vs 26 months, *P* = 0.10). No trend was observed in the other two Ki-67 groups, but the progression events were too few for any meaningful comparison.

### Dose escalation of SSA

In 127 patients, the original SSA dose was increased to a higher dose during their follow-up time because of disease progression or inadequate symptom control; of those, 47 had a second dose escalation. Dose escalation occurred concomitantly with other active treatments in 23 cases, which were therefore excluded from analysis. PFS was 9 months at first dose escalation and 6 months at second dose escalation. Dose escalation occurred for either radiological PD (*n* = 76) or biochemical PD only (*n* = 22) or because of inadequate symptom control, as per the treating doctor’s discretion (*n* = 27). PFS differed significantly depending on the reason of dose escalation (6, 9 and 22 months, *P* = 0.007)

### Treatment efficacy and Ki-67

**Figure 3:**
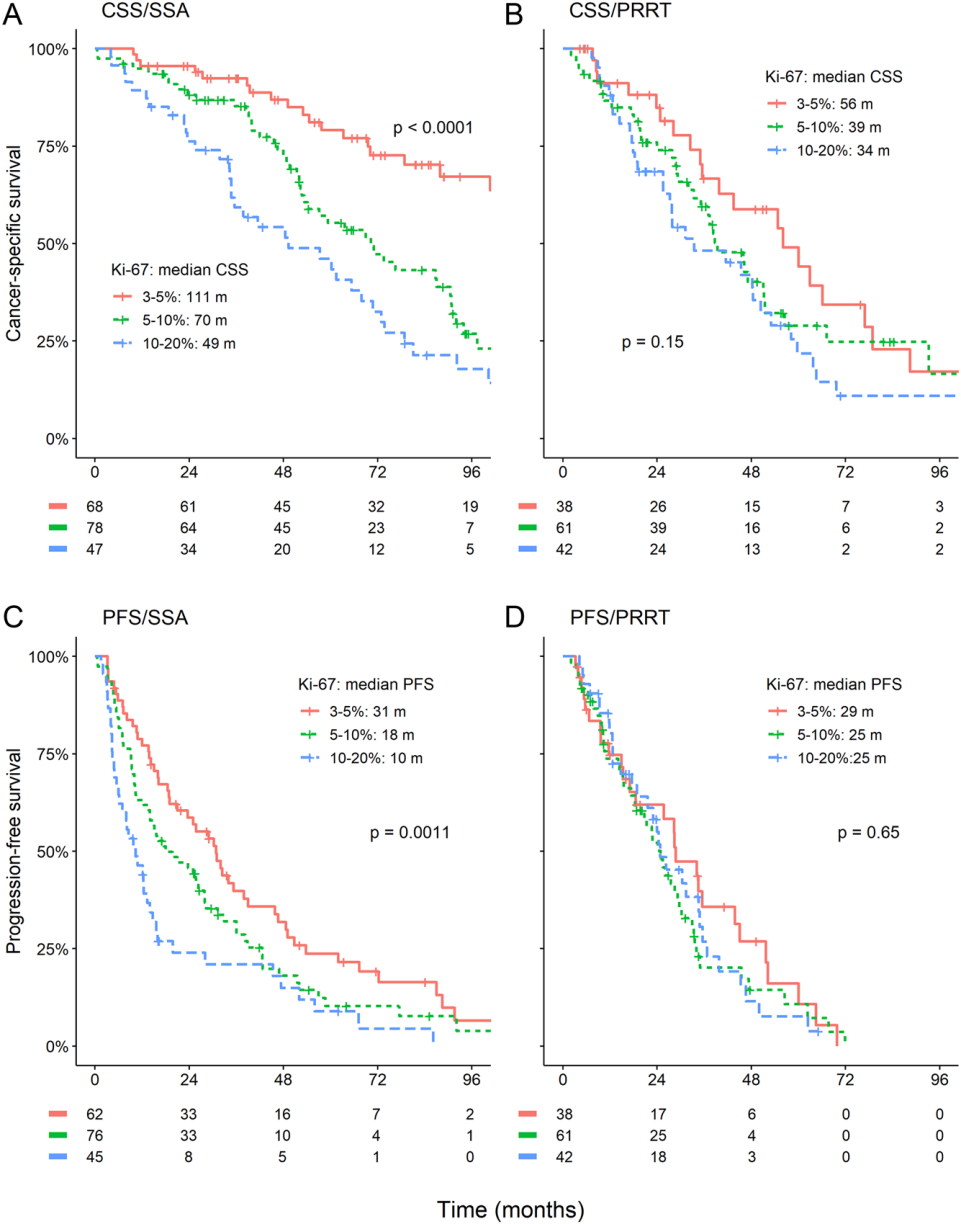
Cancer-specific (CSS) and progression-free survival (PFS) for somatostatin analogues (SSA) and peptide receptor radionuclide treatment (PRRT) by Ki-67 at 5% and 10% cut-offs. Efficacy of treatment with SSA but not with PRRT seems to diminish with increasing Ki-67. A full colour version of this figure is available at https://doi.org/10.1530/ERC-22-0316.

CSS and PFS were analysed according to Ki-67 subgroups for treatment with SSA and PRRT (respective subgroups for everolimus, chemotherapy and single IFNα were too small for meaningful interpretation). Median CSS for first-line treatment with SSA in the 3–5%, 5–10% and 10–20% subgroup was 111, 70 and 49 months, respectively. Median PFS was 31, 18 and 10 months, respectively. In the case of PRRT, differences between the Ki-67 subgroups were minimal, with respective median CSS of 56, 39 and 34 months and PFS of 29, 25 and 25 months (Fig. 3).

The relationship between survival outcomes and Ki-67 was explored assuming non-linearity in a cox regression analysis for SSA and PRRT. Curves followed in both cases a near-logarithmic transformation. For SSA, increased Ki-67 resulted in significantly higher risk for death (*P* < 0.001) and progression (*P* < 0.001) throughout the 3–20% range. For PRRT, there was only a slight increase in the risk of disease progression with increasing Ki-67, but this was not statistically significant and seemed to reach a plateau at approximately 7%.

### Treatment efficacy in relation to SSTR status

Among patients treated with single SSA, single IFNα or their combination, and in whom SSTR status was available, 13/111, 6/26 and 8/63, respectively, had low or negative SSTR status on either scintigraphy or PET imaging. In this group of patients with low or negative SSTR status, median CSS was lower for patients starting treatment with SSA alone (24 vs 74 months, *P* < 0.001) or in combination with IFNα (42 vs 106 months, *P* = 0.014), compared to the group with high SSTR expression. PFS was significantly shorter for patients with low or negative SSTR status starting treatment with single SSA (5 vs 14 months, *P* < 0.001) but not for those treated with the combination of SSA and IFNα (15 vs 32 months, *P* = 0.54). There was no significant difference for patients treated with single IFNα, either for CSS (34 vs 48 months, *P* = 0.06) or for PFS (16 vs 6 months, *P* = 0.57), when comparing SSTR positive and negative patients.

**Figure 4:**
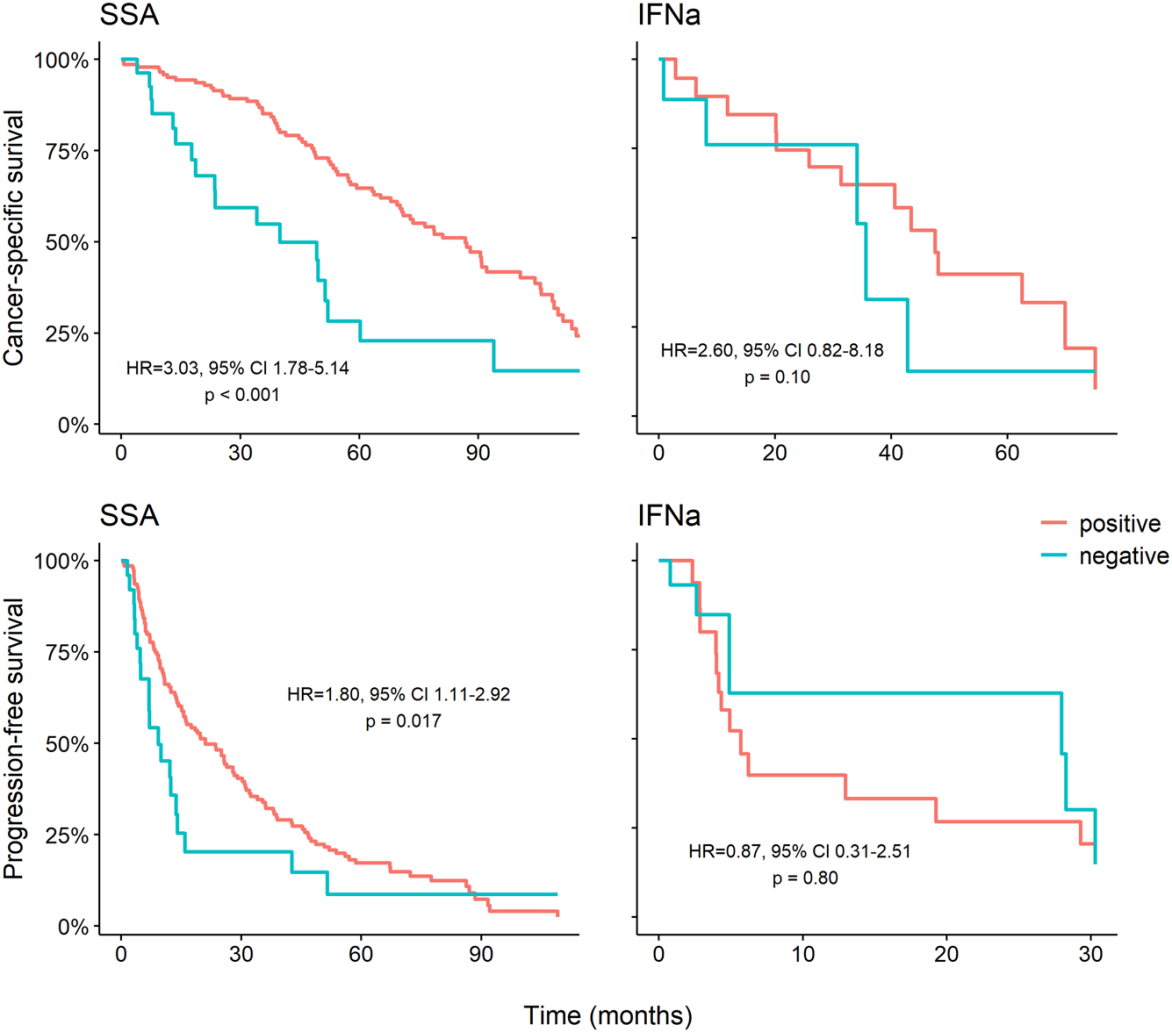
Cancer-specific and progression-free survival by somatostatin receptor (SSTR) status for patients treated with somatostatin analogues (SSA) as monotherapy or combination treatment and with interferon-alpha (IFNα), after adjusting for the difference in Ki-67 in a cox model. HR, hazard ratio; CI, confidence interval. A full colour version of this figure is available at https://doi.org/10.1530/ERC-22-0316.

Patients with low or negative SSTR status had higher Ki-67 (mean Ki-67 10.8 vs 7.4%, *P* = 0.006). Assuming that this might account for the difference in survival times, we corrected for Ki-67 in a cox regression analysis examining separately patients treated with SSA (single or in combination) and with IFNα monotherapy. The adjusted risks for cancer-related death (HR = 3.03, 95% CI 1.78–5.14, *P* < 0.001) and for disease progression (HR = 1.80, 95% CI 1.11–2.92, *P* = 0.017) remained significantly higher for patients treated with SSA in the low or negative SSTR group. Furthermore, they remained non-significant for patients treated with IFNα (HR = 2.60, 95% CI 0.82–8.18, *P* = 0.10 and HR = 0.87, 95% CI 0.31–2.51, *P* = 0.80, respectively). Adjusted for Ki-67 survival curves are shown in Fig. 4.

**Figure 5:**
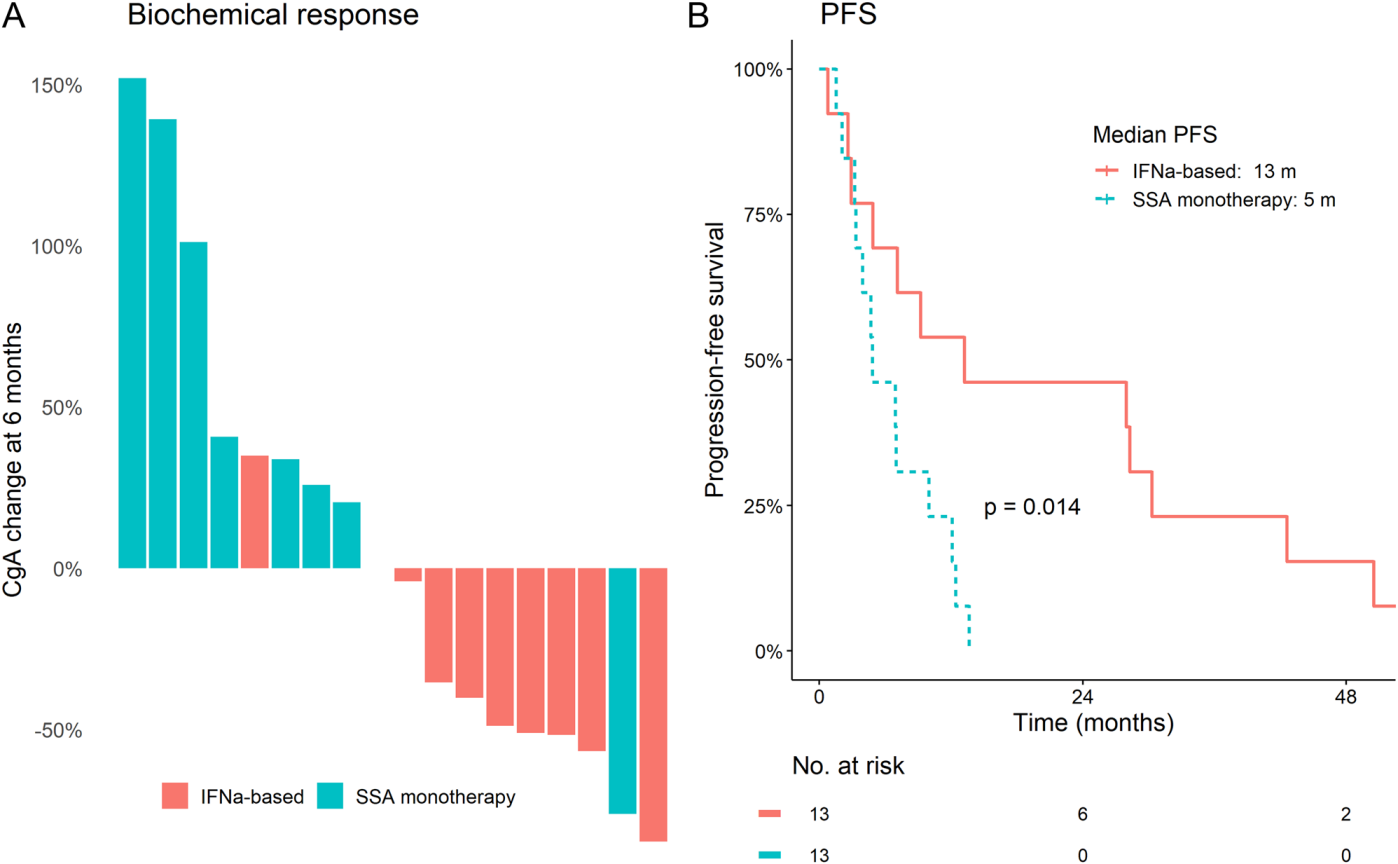
(A) Reduction of chromogranin A (CgA) was seen almost exclusively in tumours with low/negative somatostatin receptor expression, treated with interferon-alpha (IFNα), single or in combination, but not in those treated with somatostatin analogues (SSA). (B) Progression-free survival was significantly longer for patients treated with IFNα (single or in combination with SSA) compared to patients treated with only SSA. A full colour version of this figure is available at https://doi.org/10.1530/ERC-22-0316.

We further compared the efficacy of IFNα and SSA in patients with low or negative SSTR status. Biochemical stabilization or response was achieved almost exclusively in patients treated with IFNα, single or in combination (Fig. 5A). Assuming a low SSA efficacy in this group, we grouped combination patients together with single IFNα. Median PFS was significantly longer for patients treated with IFNα (13 vs 5 months, *P* = 0.014, Fig. 5B). Result was similar when excluding combination patients (16 vs 5 months, *P* = 0.014). However, the small number of patients precludes any firm conclusions.

### Additional medical treatments

Ninety-five patients received at least one injection of IFNα. Median CSS, OS and PFS for those starting IFNα in combination with SSA as first-line treatment were 105, 97 and 32 months, respectively. PFS after adding IFNα, mostly in second line, was 6 months. In 35 cases (43%), treatment was stopped because of side effects. Twenty-nine patients were treated with everolimus, mostly at later lines, with a median PFS of 5 months. Toxicity resulted in treatment discontinuation in 12 cases (41%). Seventeen patients were treated with chemotherapy, mostly at first line. The most common regimen was temozolomide single or in combination with capecitabine. Median PFS was 9 months. One hundred and sixteen patients were treated with PRRT, with a median PFS of 30 months after initial treatment (47, 30 and 19 months at first, second and ≥third line, respectively) and 13 months after rechallenge (13 and 8 months at ≤third and ≥fourth line, respectively). Among the 17 patients who had not received another treatment between initial and repeat PRRT treatment, PFS from initial PRRT was 62 months.

## Discussion

The present study showed the efficacy of SSA used as monotherapy or in combinations in patients with Si-NET G2. We found that median PFS on SSA treatment was 12.4 months on SSA monotherapy and 19 months for all patients, indicating that Si-NET G2 patients in general respond equally well to SSA as those with G1 tumours. On the other hand, use of above-label SSA doses after radiological progression resulted in a modest median PFS of 6 months. Within the G2 group, we observed a significantly shorter median PFS at higher Ki-67 levels for treatment with SSA but not with PRRT. Additionally, we noted that CSS after treatment with a higher (label) SSA starting dose was longer only for patients with Ki-67 5–10%. Finally, we show that two-thirds of patients with SSTR-negative tumours achieve at least short-term biochemical stabilization when treated with IFNα.

The PROMID trial reported a median TTP of 14.3 months in Si-NET with Ki-67 <2% (Rinke *et al.* 2009). In the CLARINET study, median PFS was not reached after 2 years, possibly because most patients had SD at baseline (Caplin *et al.* 2014). These studies used Response Evaluation Criteria in Solid Tumours, which have been shown to give 20% longer estimates of PFS in slowly growing tumours compared to conventional evaluation used in our study (Løitegård *et al.* 2019). Two retrospective Si-NET series reported a median OS of 84–104 months in treatment-naïve, predominantly G1 patients (Laskaratos *et al.* 2018, Maurer *et al.* 2022). A recent Surveillance, Epidemiology and End Results (SEER) database analysis showed a modest median survival of 41 months for metastatic Si-NET and showed that Si-NET G2 tumors were associated with a 45% higher risk for death compared to G1 tumours (Shah *et al.* 2019). In our study, median CSS of 77 months from first-line SSA was slightly shorter than all-grade Si-NET cohorts, possibly due to the more aggressive nature of G2 tumours. Efficacy seemed to be similar in PS 0–1 patients. Our median PFS of 12.4 months with first-line SSA monotherapy was similar to that in PROMID, thus confirming SSA activity also in this group with higher proliferation index.

Historically, treatment was initiated with below-label doses of SSA. PROMID and CLARINET established 30 mg octreotide LAR/120 mg lanreotide autogel every 4 weeks as standard dose. Furthermore, retrospective studies and the prospective CLARINET FORTE trial examine the efficacy of even higher SSA doses (Rinke *et al.* 2009, Ferolla *et al.* 2012, Caplin *et al.* 2014, Lamberti *et al.* 2020, Diamantopoulos *et al.* 2021, Pavel *et al.* 2021). Although SSA discontinuation for high-grade toxicity is rare, the incidence of low-grade toxicity is significant (Sorbye *et al.* 2020), with adverse events in prospective trials ranging from 31 to 51% (Caplin *et al.* 2014, Wolin *et al.* 2015, Strosberg *et al.* 2017, Pavel *et al.* 2021). Two recent cost-effectiveness analyses (representative of US prices) showed a high cost per quality-adjusted life year in some situations and are indicative of the financial burden of SSA treatment (Joish *et al.* 2018, Rustgi *et al.* 2021). We thus examined whether lower SSA doses might be equally effective. In our cohort, below-label starting SSA doses resulted in inferior CSS in patients with Ki-67 5–10% (*P* = 0.002), but we could detect no CSS difference in the subgroup of patients with lower Ki-67 (*P* = 0.54). This might signify that below-label doses are adequate for slow-proliferating tumours.

Dose escalation of SSA is often a first step in treatment intensification. Dose escalation to above-label doses of SSA has been used in the control arms of two randomized trials, reporting median PFS of 6.8 and 8.4 months (Wolin *et al.* 2015, Strosberg *et al.* 2017), almost identical to the 8.3 months for patients with progressive Si-NET in the CLARINET FORTE study (Pavel *et al.* 2021). Previous retrospective studies have reported unexpectedly high PFS of 16–31 months (Ferolla *et al.* 2012, Lamberti *et al.* 2020, Diamantopoulos *et al.* 2021). Our PFS of 6 months for patients with documented radiological PD was more in line with the prospective study results. The slightly shorter duration might reflect the lower first escalation dose (typically to label dose every 3 weeks compared to every 2 weeks in reported trials), the difference in response evaluation criteria or the higher tumour grade. Of interest, radiological follow-up occurred at 3- to 6-month intervals, and a median PFS of 6 months with 1-year PFS of 37% represents a rather modest gain.

Few studies have examined the effect of various Ki-67 cut-offs on SSA and PRRT treatment efficacy in Si-NET (Palazzo *et al.* 2013, Ezziddin *et al.* 2014, Faggiano *et al.* 2016, Aalbersberg *et al.* 2019), and none compared different treatments in the same population. We found PFS, CSS and OS to be significantly worse for SSA at the higher Ki-67 levels, whereas PRRT efficacy seemed to be largely independent of Ki-67 in the range of 3–20%, indicating a previously poorly described difference in the significance of Ki-67 for the two main treatments used nowadays. SSA, a primarily cytostatic treatment, is probably less effective against rapidly proliferating tumours, in contrast to a cytotoxic treatment such as PRRT. Indeed, in the NETTER-1 trial, PRRT was efficient for both G1 and G2 tumours, with similar HRs for PFS (0.15 and 0.24, respectively) (Strosberg *et al.* 2017). Limited data point to PRRT efficacy even in G3 NEN with high SSTR expression (Lithgow *et al.* 2021), which is consistent with the early plateau we observed in the relationship between Ki-67 and PFS. On the other hand, pivotal SSA studies included only patients with Ki-67 <10%, and small series indicate minimal effect in patients with G3 tumours (McGarrah *et al.* 2020, Lithgow *et al.* 2021, Merola *et al.* 2021). Interestingly, the European Society of Medical Oncology guidelines propose the use of everolimus before PRRT in G2 patients with Ki-67 >10% (Pavel *et al.* 2020). As only few patients were treated with everolimus, and mostly at later lines, we could not formally compare everolimus and PRRT; however, our data do not suggest any significant efficacy drop for PRRT in patients with Ki-67>10%, which could support this recommendation.

Tumours with low SSTR expression tend to have worse outcomes and limited treatment options (Refardt *et al.* 2020). In our cohort, PFS and CSS in those tumours were significantly lower for first-line treatment with SSA, single or in combinations, but did not differ significantly for patients treated with IFNα, even after correcting for the higher proliferation index of tumours with low SSTR expression. Of note, two-thirds of all patients treated with IFNα had at least short-term biochemical stabilization. IFNα might be considered as a treatment option in this population, if available.

The study has several limitations, mainly related to its retrospective nature, potential selection bias, non-standardized tumour response evaluation and cases of missing progression data. Even though Ki-67 was not re-evaluated specifically for the purpose of this study, the vast majority of cases were reviewed by dedicated NET pathologists as part of clinical routine. The study included only G2 patients, meaning that our assumptions for relation between Ki-67 levels and treatment efficacy might not be applicable outside the 3–20% range. Additionally, treatment patterns changed throughout the study. However, PRRT was used as early as 2006 and therefore an option for most patients and the use of everolimus remains infrequent. No other major treatment breakthroughs occurred during the study period. One of the major strengths of this study compared to other publications is its focus exclusively on a previously poorly described homogeneous population, allowing for representative conclusions upon treatment efficacy in this group.

## Conclusion

Treatment with SSA is effective in Si-NET G2, a group not previously studied separately. Treatment benefit from SSA depended on Ki-67 levels, whereas Ki-67 effect on PRRT efficacy was less pronounced. SSA dose intensification because of radiologically confirmed PD provided only short-term disease stabilization in this population.

## Declaration of interest

The authors declare no conflict of interest that could be perceived as prejudicing the impartiality of the research reported.

## Funding

This study was supported by the Swedish Cancer Society (18 0576) and Futurum – the Academy for Health and Care, Region Jönköping County.
